# 2020 WHO guidelines on physical activity and sedentary behaviour for children and adolescents aged 5–17 years: summary of the evidence

**DOI:** 10.1186/s12966-020-01037-z

**Published:** 2020-11-26

**Authors:** Jean-Philippe Chaput, Juana Willumsen, Fiona Bull, Roger Chou, Ulf Ekelund, Joseph Firth, Russell Jago, Francisco B. Ortega, Peter T. Katzmarzyk

**Affiliations:** 1 Healthy Active Living and Obesity Research Group, Children’s Hospital of Eastern Ontario Research Institute, 401 Smyth Road, Ottawa, Ontario Canada; 2 Department of Pediatrics, University of Ottawa, Ontario, Ontario Canada; 3 Physical Activity Unit, Department for Health Promotion, World Health Organization, Geneva, Switzerland; 4 Department of Medical Informatics and Clinical Epidemiology, Oregon Health & Science University, Portland, Oregon USA; 5 Department of Sport Medicine, Norwegian School of Sport Science, Oslo, Norway; 6 Department of Chronic Diseases and Ageing, Norwegian Institute of Public Health, Oslo, Norway; 7 Division of Psychology and Mental Health, University of Manchester, Manchester, UK; 8 NICM Health Research Institute, Western Sydney University, Westmead, Australia; 9 Centre for Exercise, Nutrition & Health Sciences, School for Policy Studies, University of Bristol, Bristol, UK; 10 The National Institute for Health Research Applied Research Collaboration West (NIHR ARC West) at University Hospitals Bristol NHS Foundation Trust, Bristol, UK; 11 PROFITH “PROmoting FITness and Health through physical activity” research group, Department of Physical Education and Sports, Faculty of Sport Sciences, University of Granada, Granada, Spain; 12 Pennington Biomedical Research Center, Baton Rouge, Louisiana USA

**Keywords:** Public health, Recommendations, Guidelines, Physical activity, Sedentary, Exercise, Policy, Youth

## Abstract

**Background:**

The World Health Organization (WHO) released in 2020 updated global guidelines on physical activity and sedentary behaviour for children, adolescents, adults, older adults and sub-populations such as pregnant and postpartum women and those living with chronic conditions or disabilities.

**Objective:**

To summarize the evidence on the associations between physical activity, sedentary behaviour, and health-related outcomes used to inform the 2020 WHO guidelines on physical activity and sedentary behaviour for children and adolescents aged 5–17 years.

**Methods:**

The update of the WHO guideline recommendations for children and adolescents utilized and systematically updated the evidence syntheses on physical activity and sedentary behaviour conducted for the 2016 Canadian 24-Hour Movement Guidelines for Children and Youth, the 2019 Australian 24-Hour Movement Guidelines for Children and Young People (5–17 years), and the 2018 Physical Activity Guidelines for Americans, Second Edition. Systematic reviews published from 2017 up to July 2019 that addressed the key questions were identified, and the Grading of Recommendations Assessment, Development and Evaluation (GRADE) framework was used to rate the certainty of the evidence for the entire body of evidence.

**Results:**

The updated literature search yielded 21 relevant systematic reviews. The evidence base reviewed (i.e., existing and new systematic reviews) provided evidence that greater amounts and higher intensities of physical activity as well as different types of physical activity (i.e., aerobic and muscle and bone strengthening activities) are associated with improved health outcomes (primarily intermediate outcomes). There was sufficient evidence to support recommendations on limiting sedentary behaviours, which was not addressed in the 2010 WHO guidelines. However, there is still insufficient evidence available to fully describe the dose-response relationships between physical activity or sedentary behaviour and health outcomes, and whether the associations vary by type or domain of physical activity or sedentary behaviour.

**Conclusions:**

Addressing the identified research gaps will better inform guideline recommendations in children and adolescents, and future work should aim to prioritize these areas of research. In the meantime, investment and leadership is needed to scale up known effective policies and programs aimed at increasing activity in children and adolescents.

## Introduction

Physical activity is well-known to provide multiple health-related benefits in children and adolescents [1]. However, 81% of adolescents aged 11–17 years are insufficiently physically active globally, with significant differences in the prevalence of insufficient physical activity across genders, regions, and countries [2]. Physical inactivity is a serious threat to the health and wellbeing of the population [3–5], and urgent scaling up of known effective policies and programs to increase population levels of physical activity, including children and adolescents, is needed [6].

Sedentary behaviour is an important consideration alongside physical activity when examining the contribution of both behaviours to the health of children and adolescents [7]. Sedentary behaviour is defined as any waking behaviour characterized by an energy expenditure ≤1.5 metabolic equivalents while in a sitting, reclining or lying posture [8]. Common sedentary behaviours include smartphone/tablet use, TV viewing, video game playing, computer use, driving or riding in a car, and reading/studying while sitting. Excessive sedentary time is widespread among children and adolescents around the world [9], and there is emerging evidence on the negative health effects and the potential public health burden associated with high levels of sedentary behaviour [10].

The World Health Organization (WHO)‘s *Global action plan on physical activity 2018–2030* [11] was launched in 2018 and all 194 WHO Member States agreed to the new target of a 15% relative reduction in physical inactivity globally by 2030, and called upon WHO to update the WHO 2010 *Global recommendations on physical activity for health* [1]. In 2019, WHO released *Guidelines on physical activity, sedentary behaviour and sleep for children under 5 years of age* [12]. In 2020, WHO released global guidelines on physical activity and sedentary behaviour for children, adolescents, adults, older adults and sub-populations such as pregnant and postpartum women and those living with chronic conditions or disabilities [13, 14].

A Guideline Development Group was established by WHO in 2019 consisting of 28 experts from relevant scientific disciplines as well as practitioners and decision makers in the field with representation from all regions to contribute to the process of developing these guidelines [13, 14]. Extensive work was undertaken during 2019–2020 to review the available evidence and formulate specific draft recommendations for consideration by WHO. The purpose of this article is to summarize the evidence on the associations between physical activity, sedentary behaviour, and health-related outcomes that was used to inform the 2020 WHO guideline recommendations on physical activity and sedentary behaviour for children and adolescents aged 5–17 years.

## Methods

The WHO guidelines were developed in accordance with the WHO Handbook for Guideline Development [15] and details of the methodology can be found elsewhere [13, 14]. The PI/ECO (Population, Intervention/Exposure, Comparison, Outcome) questions of interest for the population of children and adolescents are shown in Table 1. The population of interest was apparently healthy children and adolescents aged from 5 to under 18 years of age. A total of 9 health outcomes were chosen based on the literature, expert input and consensus, and recognizing the importance of including a broad range of outcomes (Table 2). The assessment utilized and systematically updated recent relevant evidence reviews:

- A systematic review of the literature conducted by Poitras et al. [16] on the association between physical activity and health indicators in school-aged children and adolescents as part of the process for developing the *Canadian 24-Hour Movement Guidelines for Children and Youth* [17]. This review focused only on studies that used objective or device-based measurements of physical activity. A total of 162 studies were included (204,171 participants from 31 countries) in the review.
- A systematic review of the literature on the association between sedentary behaviour and health indicators in school-aged children and adolescents conducted by Carson et al. [18] as part of the process for developing the *Canadian 24-Hour Movement Guidelines for Children and Youth* [17]. A total of 235 studies (194 unique samples) were included representing 1,657,064 unique participants from 71 different countries.
- A systematic review conducted by Okely et al. [19] undertaken to update the Poitras et al. [16] and Carson et al. [18] systematic reviews as part of the development of the 2019 *Australian 24-Hour Movement Guidelines for Children and Young People* (5–17 years) [20]. This report identified an additional 42 studies on physical activity and 32 studies on sedentary behaviour published through to July 2018. The GRADE tables developed from these updates were used as the basis for the commissioned update conducted for WHO. The GRADE tables along with the evidence profiles are presented in Annex A1 and A2 of the main WHO guideline document [13].
- The scientific report of the Physical Activity Guidelines Advisory Committee (PAGAC) [21] which provides a summary of a systematic update of evidence on physical activity and sedentary behaviours and health outcomes since 2008 through to 2016 to inform the development of the 2018 *Physical Activity Guidelines for Americans, 2nd Edition* [22].

**Table 1:** Questions related to physical activity and sedentary behaviour in children and adolescents aged 5–17 years that were addressed by the WHO Youth Working Group

*Physical activity questions*	
1. What is the association between physical activity and health-related outcomes?	
2. Is there a dose-response association (volume, duration, frequency, intensity)?	
3. Does the association vary by type or domain of physical activity?	
*Sedentary behaviour questions*	
1. What is the association between sedentary behaviour and health-related outcomes?	
2. Is there a dose-response association (total volume and the frequency, duration and intensity of interruptions)?	
3. Does the association vary by type or domain of sedentary behaviour?

**Table 2:** List of critical and important outcomes chosen by expert agreement among the WHO Guideline Development Group for children and adolescents aged 5–17 years

Outcomes	Importance
Physical fitness (e.g., cardiorespiratory, motor development, muscular fitness)	Critical
Cardiometabolic health (e.g., blood pressure, dyslipidemia, glucose, insulin)	Critical
Bone health	Critical
Adiposity	Critical
Adverse effects (e.g., injuries and harms, respiratory effects of air pollution)	Critical
Mental health (e.g., depressive symptoms, self-esteem, anxiety symptoms, ADHD)	Critical
Cognitive outcomes (e.g., academic performance, executive function)	Critical
Prosocial behaviour (e.g., conduct problems, peer relations, social inclusion)	Important
Sleep duration and quality	Important

An external team of reviewers independently assessed the methodological quality of each systematic review using the A MeaSurement Tool to Assess systematic Reviews (AMSTAR) 2 rating scale [23], and assigned an overall grade of “high”, “moderate”, “low” and “critically low” quality. The Grading of Recommendations Assessment, Development and Evaluation (GRADE) framework was used to rate the certainty of the evidence [24]. GRADE categorizes the quality of evidence into four groups (“high”, “moderate”, “low”, and “very low”). More details can be found elsewhere [13, 14]. The evidence profiles were reviewed by the Guideline Development Group and an assessment of the evidence supporting the recommendations was conducted.

## Results

### WHO update for children and adolescents aged 5–17 years

A total of 21 systematic reviews, published from 2017 to July 2019, that examined the association between physical activity and/or sedentary behaviour and the selected health outcomes were found [25–45]. Of note, the term “health outcomes”, as used in this manuscript, is meant to include both intermediate outcomes (e.g., adiposity, blood lipids, blood pressure, fitness) and clinical outcomes (e.g., cognition, mood, quality of life, cardiovascular events). Overall, 14 reviews examined the association between physical activity and health outcomes, 5 reviews examined the association between sedentary behaviour and health outcomes, and 2 reviews included both physical activity and sedentary behaviour (Table 3). Adiposity (9 reviews), cardiometabolic health (5 reviews) and cognitive outcomes (4 reviews) were the most reported outcomes in the reviews.

**Table 3:** List of systematic reviews included in the WHO search update

Author, Year	Behaviour		Outcomes										
	PA	SB	Physical fitness	CM health	Bone health	Adiposity	AEs	Mental health	Cognitive outcomes	Prosocial behaviour	Sleep duration and quality	Number of included studies	AMSTAR 2 rating
Bea, 2017 [25]	X			X								13	Moderate
Belmon, 2019 [26]		X									X	45	Low
Cao, 2019 [27]	X		X									17	Low
Collins, 2018 [28]	X					X						18	Low
Eddolls, 2017 [29]	X			X		X						13	Low
Errisuriz, 2018 [30]	X		X			X						12	Critically Low
Fang, 2019 [31]		X				X						16	Low
Koedijk, 2017 [32]		X			X							17	Moderate
Krahenbühl, 2018 [33]	X				X							21	Critically Low
Lee, 2018 [34]	X					X						27	Critically Low
Marker, 2019 [35]		X				X						24	Low
Marques, 2018 [36]	X								X			51	Moderate
Martin, 2017 [37]	X					X			X			15	Moderate
Miguel-Berges, 2018 [38]	X					X						36	Low
Mohammadi, 2019 [39]	X	X				X						17	Low
Pozuelo-Carrascosa, 2018 [40]	X			X								19	Moderate
Singh, 2019 [41]	X								X	X		58	Critically Low
Skrede, 2019 [42]	X	X		X								30	Critically Low
Stanczykiewicz, 2019 [43]		X						X				31	Low
Verswijveren, 2018 [44]	X			X								29	Moderate
Xue, 2019 [45]	X								X			19	Low

Table produced by WHO and part of the Evidence Profiles available as a web annex to the main guideline document [13]

Abbreviations: *AEs* adverse effects, *CM* cardiometabolic, *PA* physical activity, *SB* sedentary behaviour

No new reviews examined the association between physical activity and adverse effects, mental health or sleep and no new reviews examined the association between sedentary behaviour and physical fitness, adverse effects, cognition or prosocial behaviour. None of the new reviews directly examined whether there was a dose-response association between physical activity or sedentary behaviour and health outcomes, or whether the association varied by type or domain of physical activity or sedentary behaviour. In most cases, each review was narrowly scoped to examine specific intensities or intervention programs aimed at increasing physical activity (e.g., high-intensity interval training, school-based physical activity programs) or sedentary behaviour (e.g., device-measured sedentary time), and was limited to specific study designs (e.g., only randomized controlled trials).

None of the new systematic reviews were rated as having high credibility based on AMSTAR 2 criteria. Six were rated as having moderate credibility and 10 were rated as having low credibility. Five reviews were rated as having critically low credibility and were thus excluded from the final Evidence Profiles. The full Evidence Profile tables for children and adolescents are available as a web annex to the main guideline documents [13, 14]. For both physical activity and sedentary behaviour, the summary of evidence below refers to the overall body of evidence from existing [16, 18, 19, 21] and the 21 new systematic reviews.

### Summary of evidence for physical activity in children and adolescents

#### Question 1: what is the association between physical activity and health outcomes?

The evidence shows that greater amounts and higher intensities of physical activity are associated with multiple beneficial health outcomes, including cardiorespiratory fitness, muscular fitness, bone health, and cardiometabolic health. The evidence also shows that physical activity reduces the risk of experiencing depression, and that physical activity interventions reduce depressive symptoms in children and adolescents with and without major depression. Physical activity has positive effects on cognitive function and academic outcomes (e.g., school performance, memory and executive function) in children and adolescents. Physical activity is also favourably associated with adiposity in children and adolescents. Null findings were generally observed in the studies that examined the association between physical activity and motor skill development. There is no or very limited evidence available to conclude on the association between physical activity and adverse effects, prosocial behaviour or sleep. The overall quality of the evidence for physical activity was rated as “moderate” according to GRADE.

#### Question 2: is there a dose-response association (volume, duration, frequency, intensity)?

The shape of the dose-response curve and/or the presence of threshold values that can differentiate lower versus higher risk in the associations between physical activity and health outcomes is poorly understood in children and adolescents. Although the optimal physical activity dose associated with improved health outcomes cannot be determined precisely with the available evidence base, many of the benefits are observed with an average of 60 min of moderate-to-vigorous intensity physical activity (MVPA) daily, although more physical activity beyond 60 min of MVPA daily appears to be better for various health outcomes.

#### Question 3: does the association vary by type or domain of physical activity?

There is currently insufficient evidence to determine if the association between physical activity and health outcomes varies by type (e.g., aerobic vs. strength-promoting exercise) or domain of physical activity (e.g., active transport [walking and cycling] vs. physical education vs. sports/recreation) in children and adolescents. There is evidence showing that increased aerobic MVPA increases cardiorespiratory fitness and that increased resistance exercise increases muscular fitness in children and adolescents, with some evidence showing incremental benefits of doing both. The evidence suggests that incorporating muscle and bone strengthening activities at least 3 days per week is beneficial. However, there is a lack of evidence on other characteristics of physical activity, such as the duration of activities that strengthen muscles and bones, to be able to add a duration to the frequency component. There is also insufficient evidence that would suggest there are different health benefits from different domain-specific physical activities (e.g., active transportation vs. physical education vs. sports/recreation). Thus, future research should aim to address this important knowledge gap as it relates to “type” and “domain” of physical activity in children and adolescents to help provide more guidance and specificity to this component of the guidelines.

### 2020 WHO guidelines in the context of previous physical activity guidelines

The 2020 WHO guidelines call for children and adolescents to accumulate at least an average of 60 min of MVPA per day (mostly aerobic physical activity). They also recommend that vigorous physical activities and muscle and bone strengthening activities should each be incorporated at least 3 days per week. This new guideline is similar to physical activity guidelines from most countries (e.g., Canada [17], Australia [20], USA [22], UK [46]) and in line with the previous WHO physical activity guidelines released in 2010 [1]. However, it is now explicit in the guidelines that children can accumulate physical activity through an average of 60 min of MVPA per day and not necessarily on all 7 days of the week. As studies have broadly used an average threshold of 60 min per day, not a minimum daily threshold of 60 min, to assess the benefits of physical activity on health outcomes, the recommendation was updated in recognition of the evidence and the ways that physical activity is measured in children and adolescents [17]. Please refer to Table 4 for the 2020 WHO physical activity guidelines for children and adolescents.

**Table 4:** 2020 WHO physical activity guidelines for children and adolescents (5–17 years)

For children and adolescents, physical activity can be undertaken as part of recreation and leisure (play, games, sports or planned exercise), physical education, transportation (wheeling, walking and cycling) or household chores, in the context of educational, home, and community settings. In children and adolescents, physical activity confers benefits for the following health outcomes: improved physical fitness (cardiorespiratory and muscular fitness), cardiometabolic health (blood pressure, dyslipidaemia, glucose, and insulin resistance), bone health, cognitive outcomes (academic performance, executive function), mental health (reduced symptoms of depression); and reduced adiposity. **It is recommended that:** • Children and adolescents should do at least an average of 60 minutes per day of moderate- to vigorous-intensity, mostly aerobic, physical activity, across the week. ***Strong recommendation, moderate certainty evidence*** • Vigorous-intensity aerobic activities, as well as those that strengthen muscle and bone, should be incorporated at least 3 days a week. ***Strong recommendation, moderate certainty evidence*** ***Good practice statements:*** ❖ *Doing some physical activity is better than doing none.* ❖ *If children and adolescents are not meeting the recommendations, doing some physical activity will benefit their health.* ❖ *Children and adolescents should start by doing small amounts of physical activity, and gradually increase the frequency, intensity and duration over time.* ❖ *It is important to provide all children and adolescents with safe and equitable opportunities, and encouragement, to participate in physical activities that are enjoyable, offer variety, and are appropriate for their age and ability.*	

### Summary of evidence for sedentary behaviour in children and adolescents

#### Question 1: what is the association between sedentary behaviour and health outcomes?

There is evidence to suggest that greater time spent in sedentary behaviour, especially recreational screen time, is related to poorer health outcomes in children and adolescents. For example, higher duration of screen time (including TV viewing) is associated with lower fitness, poorer cardiometabolic health, shorter sleep duration, and unfavourable measures of adiposity. Higher durations of screen time and some aspects of computer use can be associated with poorer mental health, and TV viewing and video gaming are associated with unfavourable measures of behavioural conduct/pro-social behaviour. However, certain types of sedentary behaviour, such as reading and doing homework outside of school, are associated with higher academic achievement, indicating that there are differences depending on the activity. There is some evidence to suggest that sedentary behaviour is not related to bone health in children and adolescents. Evidence that sedentary behaviours are linked to adverse health outcomes could be the result of either direct effects of the sedentary behaviours, displacement of time spent in more physically active behaviours, or both. The overall quality of the evidence for sedentary behaviour was rated as “low” using GRADE and may reflect the ways that sedentary behaviour has been assessed and the cross-sectional nature of many of the studies.

#### Question 2: is there a dose-response association (total volume and the frequency, duration and intensity of interruptions)?

There is currently insufficient evidence available to determine whether a dose-response relationship exists between sedentary time (including recreational screen time) and health outcomes in children and adolescents. However, less time spent in sedentary behaviours appears to be better for health outcomes.

#### Question 3: does the association vary by type or domain of sedentary behaviour?

There is currently insufficient evidence available to determine if the association between sedentary behaviour and health outcomes varies by type or domain of sedentary behaviour. The association between sedentary behaviour and adverse health outcomes is generally stronger for television viewing or recreational screen time as the specific exposure variable than for total sedentary time. The committee also recognized that time spent in sedentary behaviour may include educational pursuits or quiet activities without electronic media (e.g., reading, studying, drawing, crafting, listening to music, doing puzzles). These are important for child development and have cognitive as well as other benefits. The committee acknowledged the importance of reflecting the value of sedentary time that is known to benefit cognitive function and social interaction in developing the recommendations.

### 2020 WHO guidelines in the context of previous sedentary behaviour guidelines

The 2020 WHO guidelines recommend that children and adolescents limit the amount of time spent in sedentary behaviours, and especially the amount of recreational screen time. Some national guidelines recommend limiting sedentary recreational screen time to no more than 2 h per day and recommend breaking up long periods of sitting as often as possible [17, 20, 46]. However, there is currently insufficient evidence to specify precise cut-offs for recreational screen time. Most of the evidence assessing the associations between sedentary behaviours and health outcomes in children and adolescents is cross-sectional in nature, with a majority of studies relying on self- or parent-reported measures of sedentary time that are more affected by measurement errors and recall biases. National guidelines that have defined threshold values for recommended recreational screen time despite recognized limitations in the evidence have done so in response to health care provider and public requests for more specificity on sedentary behaviour and the low potential risks. Table 5 summarises the 2020 WHO sedentary behaviour guidelines for children and adolescents.

**Table 5:** 2020 WHO sedentary behaviour guidelines for children and adolescents (5–17 years)

Sedentary behaviour is defined as time spent sitting or lying with low energy expenditure, while awake, in the context of educational, home, and community settings and transportation. In children and adolescents, higher amounts of sedentary behaviour are associated with the following poor health outcomes: increased adiposity; poorer cardiometabolic health, fitness, behavioural conduct/pro-social behaviour; and reduced sleep duration. **It is recommended that:** • Children and adolescents should limit the amount of time spent being sedentary, particularly the amount of recreational screen time. ***Strong recommendation, low certainty evidence***	

## Discussion

The main purpose of this paper was to briefly describe the updated scientific evidence that informed the new 2020 WHO guideline recommendations for children and adolescents aged 5–17 years and to summarize the key research gaps in this field. A key difference between the 2010 and the 2020 WHO guidelines is the inclusion of sedentary behaviour guideline recommendations. However, there remain important research gaps. Future work should aim to prioritize these areas of research to better inform public health guidelines (Table 6). In the meantime, investment and leadership is needed to scale up known effective policies and programs aimed at increasing activity in children and adolescents.

**Table 6:** List of key research gaps to be addressed to better inform future physical activity and sedentary behaviour guideline recommendations in children and adolescents aged 5–17 years

1. Research is needed to develop standardized and harmonized methods of processing device-based measures of physical activity and sedentary behaviour.	
2. Randomized controlled trials and prospective cohort studies that use device-based measures are needed to elucidate the causal and independent dose-response associations between physical activity or sedentary behaviour and health outcomes.	
3. Work is needed to better address whether the associations between physical activity or sedentary behaviour and health outcomes vary by type or domain of physical activity or sedentary behaviour.	
4. More work needs to examine the interactive effects of physical activity and sedentary behaviour on health outcomes. It is possible that higher levels of physical activity may be needed among youth who spend large amounts of time in sedentary behaviours.	
5. Studies that examine the effects of newer forms of sedentary behaviour (e.g., smartphones, tablets) on various health outcomes are needed as well as studies that try to determine the role of interruptions or breaks in sedentary behaviour (e.g., quantifying the optimal combination of frequency, intensity and duration of interruptions).	
6. Future studies should include a broader range of outcomes when examining the association between physical activity or sedentary behaviour and health (e.g., mental health, cognition, academic achievement, quality of life, motor skill development, and musculoskeletal outcomes such as spine/neck problems associated with screen use).	
7. Future studies will need to conduct subgroup analyses to determine whether the patterns of association between physical activity or sedentary behaviour and health outcomes vary by age, sex, race/ethnicity, socioeconomic status and/or weight status. This knowledge gap substantially limits the ability to determine whether guideline recommendations should be applied broadly to the population or adapted to specific subgroups.	

The evidence reviewed on the associations between physical activity and health outcomes in children and adolescents reaffirms the findings reported in the 2010 WHO guidelines [1]. The volume and quality of the evidence base to support the guideline recommendations have markedly increased in the past 10 years. The evidence base is now more robust and supports the conclusions that greater amounts and higher intensities of physical activity as well as different types of physical activity (i.e., aerobic and muscle and bone strengthening activities) are associated with improved health outcomes (primarily intermediate outcomes). The change from recommending that children do at least 60 min of physical activity per day to at least an average of 60 min per day reflects the evidence on the association between MVPA and improved health outcomes and the way MVPA has been measured. This will have implications for future surveillance of physical activity in this age group.

Evidence to determine a precise dose-response association or variation by type or domain of physical activity or sedentary behaviour is still lacking. This highlights an important research gap that should be addressed in future studies. Furthermore, researchers are encouraged to examine a broader set of outcomes because the most reported outcomes in the reviews examined were adiposity and cardiometabolic health. Some of the key outcomes should include quality of life and mental health, and longer-term effects on cardiovascular health should be examined.

The 2020 WHO guidelines include limiting sedentary behaviour as a separate and distinct recommendation. This evolution of public health guidelines is supported by the emerging body of evidence on the negative health effects and potential public health burden associated with high levels of sedentary behaviour in the population [7, 9, 10, 18]. However, there remain important knowledge gaps to be addressed in future studies of the field of sedentary behaviour (see Table 6) that may support the development of more precise recommendations on time limits for sedentary behaviour in the future. For example, future studies in children and adolescents will need to examine whether greater amounts and higher intensities of physical activity can mitigate the detrimental effects associated with high levels of sedentary behaviour, as documented in adults [47].

Although the foundation of guideline recommendations is the best available evidence, many other important considerations come into play. Using the GRADE Evidence to Decision (EtD) framework, the recommendations and the rating of their strength (strong or conditional/weak) were based on the quality of the available evidence in addition to considerations of the balance of benefits to harms, the sensitivity to values and preferences, the potential impact on health equity, as well as acceptability, feasibility, and resource implications. The physical activity recommendation was rated strong based on moderate quality evidence that benefits would outweigh harms. The sedentary behaviour recommendation was rated strong based on low quality evidence that benefits would outweigh harms. Both recommendations were considered to be consistent with the values and preferences of the affected populations with regard to the outcomes (i.e., not preference sensitive), with potential to improve social and health equity if implemented broadly, to be acceptable, feasible and without burdensome resource implications [13, 14, 24].

## Conclusions

The 2020 WHO guideline recommendations for children and adolescents aged 5–17 years were developed using a systematic, evidence-based, and independent process. The evidence on physical activity in children and adolescents has grown considerably since the WHO 2010 *Global recommendations on physical activity for health* [1] and reaffirms the association between physical activity and health outcomes in children and adolescents. The inclusion of specific sedentary behaviour recommendations reflects the emergence of new evidence on sedentary behaviour in the past 10 years and the need to address this behaviour as a distinct entity. Both the physical activity and sedentary behaviour are strong recommendations, as the potential benefits of following these guidelines far exceed potential risks. Important research gaps remain to be addressed and should be prioritized to inform future guidelines. In the meantime, working collaboratively towards achieving these targets is needed to improve the health and well-being of children and adolescents around the world.

## Data Availability

Not applicable.

## Abbreviations

AMSTAR: A MeaSurement Tool to Assess systematic Reviews
GRADE: Grading of Recommendations Assessment, Development and Evaluation
MVPA: Moderate-to-vigorous physical activity
PAGAC: Physical Activity Guidelines Advisory Committee
PI/ECO: Population, Intervention/Exposure, Comparison, Outcome
WHO: World Health Organization
